# What are the most important tasks of tutors during the tutorials in hybrid problem-based learning curricula?

**DOI:** 10.1186/s12909-015-0368-4

**Published:** 2015-05-06

**Authors:** Ruth Boelens, Bram De Wever, Yves Rosseel, Alain G Verstraete, Anselme Derese

**Affiliations:** 1 Department of Educational Studies, Ghent University, Henri Dunantlaan 2, 9000 Ghent, Belgium; 2 Department of Data Analysis, Ghent University, Henri Dunantlaan 1, 9000 Ghent, Belgium; 3 Department of Clinical Chemistry, Microbiology and Immunology, Ghent University, De Pintelaan 185, 9000 Ghent, Belgium; 4 Department of Family Medicine and Primary Healthcare, Ghent University, De Pintelaan 185, 9000 Ghent, Belgium

**Keywords:** Problem-based learning, Tutor roles

## Abstract

**Background:**

In problem-based learning, a tutor, the quality of the problems and group functioning play a central role in stimulating student learning. This study is conducted in a hybrid medical curriculum where problem-based learning is one of the pedagogical approaches. The aim of this study was to examine which tutor tasks are the most important during the tutorial sessions and thus should be promoted in hybrid (and in maybe all) problem-based learning curricula in higher education.

**Methods:**

A student (*N* = 333) questionnaire was used to obtain data about the problem-based learning process, combined with the achievement score of the students on a multiple-choice exam. Structural equation modeling was used to test the fit of different models (two existing models and a new simplified model) representing the factors of interest and their relationships, in order to determine which tutor characteristics are the most important in the present study.

**Results:**

A new simplified model is presented, which demonstrates that stimulation of active and self-directed learning by tutors enhances the perceived case quality and the perceived group functioning. There was no significant effect between the stimulation of collaborative learning and perceived group functioning. In addition, group functioning was not a significant predictor for achievement.

**Conclusions:**

We found that stimulating active and self-directed learning are perceived as tutors’ most important tasks with regard to perceived case quality and group functioning. It is necessary to train and teach tutors how they can stimulate active and self-directed learning by students.

## Background

Earlier research has shown that important skills such as critical thinking, problem-solving, communication, collaboration, and self-regulation can be stimulated and developed by problem-based learning (PBL) [1-4]. PBL is designed to apply knowledge instead of just acquiring knowledge and has been called one of the best examples of a constructivist pedagogical approach [3,5,6]. PBL is especially recommended as a promising approach with respect to skill development and long-term retention of knowledge [7].

One of the key constructs in a typical PBL curriculum is working in small groups: five to eight students work together in a group, under the supervision of one or more tutor(s) [8]. The tutor has an important role, as authors argue that especially average students (in comparison with students who are academically stronger) may depend more on the tutor to guide and motivate them in order to achieve the learning goals [9]. The tutor has a role as the facilitator of learning [3,4] without being a primary information resource [8]. Due to the fact that the tutor has a key role when it comes to organize well-established PBL activities, the present study focuses on the activities of the tutor in the small groups or tutorial sessions [3]. In a recent review, authors synthesized studies that address group interaction in PBL [10]. In addition to the influence of the tutor (e.g. perceptions, background, group-dynamic skills) on group interaction, also two other factors are mentioned: student factors (training, perceptions, reflection, etc.) and the problem characteristics [10]. In the present study, the influence of (a) the tutor competencies and (b) the quality of the PBL problems on group functioning is investigated. The present study is focusing on a specific case of PBL tutorial groups. There are two differences between the well-known PBL activities: first, the tutorial groups under investigation in the present study are part of a hybrid PBL curriculum, meaning that there are fewer tutorials and more plenary lectures. Second, as the tutorial groups consist of about 16 students, the groups are larger than usual.

**Figure 1:**
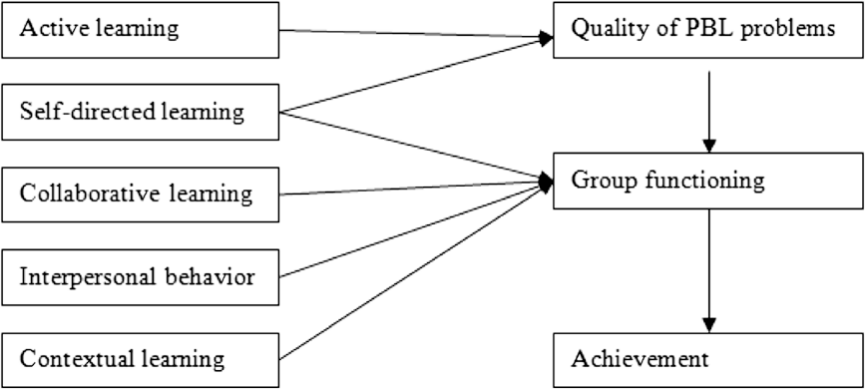
Theoretical model of van Berkel and Dolmans [5].

In a previous study, van Berkel and Dolmans investigated and tested a theoretical model (see Figure 1) that depicted the influence of different tutoring competencies on the perceived quality of PBL problems, group functioning, and student achievement in PBL [5]. The five different tutor competencies are related to how tutors deal with (a) active learning, (b) self-directed learning, (c) collaborative learning, (d) interpersonal behavior, and (e) contextual learning (see Table 1 for more details on the individual items). These five theoretical dimensions are based on constructivist approaches to learning [9,11]. Constructivist learning implies an active engagement of the learners to discover and construct knowledge on their own. Key components are self-directed learning and meaningful contexts [12].

**Table 1:** **Factors or variables and underlying items and rating scales included in the model**

Factors/variables	Individual items
Tutor competencies	
*Active learning*	The tutor stimulated us…
	1. … to summarize in our own words what we had learned
	2. … to search for links between issues discussed in the tutorial group
	3. … to understand underlying mechanisms/theories
*Self-directed learning*	The tutor stimulated us…
	4. … to generate clear learning objectives by ourselves
	5. … to search for various resources by ourselves
*Contextual learning*	The tutor stimulated us…
	6. … to apply knowledge to the problem discussed
	7. … to apply knowledge to other situations/problems
*Collaborative learning*	The tutor stimulated us…
	8. …to give constructive feedback about our group work
	9. …to evaluate group collaboration regularly
*Interpersonal behavior as tutor*	10. The tutor had a clear picture about his/her strengths/weaknesses as a tutor
	11. The tutor was clearly motivated to fulfill the role as tutor
Quality of the PBL problems	12. The problems sufficiently stimulated group discussion
	13. The problems encouraged self-study
Group functioning	14. Give a mark (1-10) for tutorial group productivity (the group always arrives at a good final result)
	15. Give a mark (1-10) for tutorial group functioning (collaboration between students)
Achievement	16. Score on a multiple-choice exam (0-10)

The relationships examined in the causal model of van Berkel and Dolmans were based on several process-oriented studies, including a causal model of PBL of Gijselaers and Schmidt [5,13]. The latter authors demonstrate the importance of well-constructed learning materials for the quality of the process of PBL and the impact of tutors’ behavior on group functioning and interest of students. The relationships examined in the causal model of van Berkel and Dolmans are also based on a study of van den Hurk et al. who focused on the quality of the learning issues, the individual study, the reporting phase and on student achievement [5,14]. The causal model (Figure 1) showed an effect from the perceived quality of the PBL problems on group functioning and an effect of group functioning on student achievement [5]. After testing the theoretical model with the data, the authors proposed a simplified theoretical model, in which the effects of two input variables (interpersonal behavior and contextual learning) were removed. Their findings suggest that stimulating active learning, self-directed learning and collaborative learning are the most important tutor tasks, resulting in higher perceived quality of the PBL problems, better group functioning, and indirectly better achievement [5].

Although the research of van Berkel and Dolmans provides a clear indication of the most important tutor competencies in view of the quality of PBL problems, group functioning, and achievement in the context of their PBL curriculum, there are two main reasons for exploring these relations in a new setting [5]. The first reason is that more data are needed to confirm the findings. This was also indicated by van Berkel and Dolmans (5, p736) themselves, as they mentioned that “in further research the model should be tested against other datasets”. The second reason is based on the differences between contexts. Strobel and van Barneveld conclude in their meta-synthesis that PBL is always implemented in particular contexts so there must be a shift from researching the effectiveness of PBL to a focus on studying the effectiveness of support structures to find optimal scaffolding, coaching, and modeling strategies for successful facilitation of PBL [7]. Indeed, according to Walker and Leary, the type of PBL implementation might play a role in learning outcomes and – as we presume – in the learning process [15]. In this respect, we especially wanted to study whether similar relationships occur in a curriculum that is more “hybrid”, i.e. in which there are fewer tutorials and more plenary lectures, as is the case in the present study [16]. This is a first difference compared with previous research conducted in settings that are completely built with PBL courses [5]. Another difference is that in our study, students meet in tutorial groups of about 16 students while in Maastricht the groups are smaller (9–10 students) [5]. More details about the hybrid curriculum can be found in the section ‘method – setting’.

Our research question was: which tasks of the tutor are most important in a hybrid PBL curriculum? More specifically, we wanted to investigate whether there are differences with full PBL curricula. Since the type of PBL implementation might play a role in the learning process and students in our curriculum have far less experience (due to the hybrid curriculum), one may expect possible differences with the results reported by van Berkel and Dolmans [5,15].

## Methods

### Setting

The study was conducted in a hybrid PBL curriculum instead of a full PBL curriculum. A short explanation of the hybrid PBL curriculum is given. The undergraduate bachelor of medical sciences curriculum (after which follows the master of medical sciences, which is equivalent to the MD degree) consists of three years and is organized in ‘blocks’, ‘threads’ and a ‘studium generale’ [17]. The ‘blocks’ form a continuum of 4 to 6 weeks where students focus on a particular theme. In addition to the blocks, there are four ‘threads’, which are running throughout all three academic years.

This study focusses on the nine tutorial sessions in the third year of the undergraduate medical curriculum that take place in the thread ‘medical problem solving and evidence based medicine’. Within this thread, no complementary lectures are given. In addition to these nine tutorials, the students have three other tutorials during the third year (as part of the blocks), but these are not included in this study. Table 2 shows the number of tutorials (and accompanying number of credits) in the third year of the undergraduate medical curriculum. It is important to notice that students had prior experience with tutorials during year 1 and 2, although to a lesser extent. In the Ghent curriculum, students have 6 tutorials in the first year, 9 tutorials in the second year and 12 tutorials in the third year.

**Table 2:** **Number of tutorials and corresponding number of credits in the third year of the undergraduate medical curriculum**

Courses	Number of tutorials	Number of credits
*‘Blocks’*		
Research Methodology	0	6
Health and Society	0	7
Concepts of clinical medicine	2	7
Concepts of clinical infectiology	1	3
Diagnostic and Therapeutic Methods	0	8
Reproduction and sexuality	0	7
Problems of Nose, Ear, Throat, Neck and Skin	0	7
*‘Threads’*		
Communication and Clinical Examination II	0	3
Medical Problem Solving and ‘Evidence Based Medicine’	9	3
Projects: Analysis and Reporting of Research Data	0	3
Exploration of youth health care. Exposure to family medicine. Studium Generale	0	6

The nine tutorials, dealing with a variety of subjects, are guided by one tutor throughout the whole year. All tutors are medical specialists who are academic faculty members and there is a tutor training for new tutors. The tutors have 1–12 years of experience with facilitating tutorial sessions. The nine tutorials (contact moments) take on average fifteen hours per year, without preparation time. In addition to facilitating tutorial sessions, the tutors have different main tasks: from the practice of the medical profession to giving lectures. Most of them are full-time clinicians and part-time professors. In their training, they are not encouraged to function as content experts, but for tutorials that are close to their expertise they might give examples or advice based on their experience and daily practice. All tutors will stop the discussion when it is going too far away from the learning objectives.

The PBL sessions are organized according to the seven steps as described by Schmidt [18]. After presenting the problem, (1) unclear terms are clarified, (2) the problem is defined, (3) the problem is analyzed, (4) a hypothesis is drawn and (5) learning objectives are established. After this first tutorial session, students have two weeks (in between lectures and other educational activities) to (6) search individually for extra information. The last step takes place in the second tutorial session: (7) synthesis and applying the new knowledge to the case.

There is a separately organized multiple-choice exam on the knowledge content of the tutorials. The open-book examination measures the application of knowledge, which is in alignment with PBL. The multiple-choice exam entails 20 to 25 questions, based on the nine tutorials, so two to three questions per tutorial. Furthermore, active participation to the tutorials was required and scored by the tutor.

### Instrument

Our research instrument, the questionnaire for students, (see Table 1) consists of 16 items. The first 11 items that measure the five characteristics of the tutor were based on a short questionnaire of Dolmans et al. that was validated in an earlier study [11,19]. Five factors are represented: active learning (items 1–3); self-directed learning (items 4 and 5); contextual learning (items 6 and 7); collaborative learning (items 8 and 9); and interpersonal behavior of the tutor (items 10 and 11). The items consist of statements and students are asked to indicate agreement on a 5-point scale (1 = strongly disagree, 5 = strongly agree). Based on the questionnaire of van Berkel and Dolmans two items were added concerning the quality of the PBL problems (items 12 and 13) on a 5-point scale [5]. The next two items measure group functioning. The first one (item 14 in our study) is based on van Berkel and Dolmans, who measured the factor group functioning with one single item [5]. Following other researchers who have measured group functioning with more items than only the group productivity, we added a second item (item 15) measuring group functioning [20,21]. Both items are based on a 10-point scale. Finally, the results on the multiple-choice exam in the third year were used to measure student achievement. The students filled in the questionnaire at the end of the academic year, after completing the nine tutorials.

### Statistical analysis

We made use of the R packages “lavaan” and “lavaan.survey” to test the model [22,23]. First, we conducted a confirmatory factor analysis (CFA) to determine whether the data fit the hypothesized measurement model. Based on this analysis, the model was found adequate. In a second step, the structural relations between the (latent) variables were studied using structural equation modeling (SEM) [24]. The following fit indices were interpreted to evaluate the different models: chi-square, Comparative Fit Index (CFI), Tucker-Lewis Index (TLI), Root Mean Square Error of Approximation (RMSEA), Standardized Root Mean Square Residual (SRMR). The data analysis was complicated by the fact that students in the same tutoring group share the same tutor. This resulted in clustered data, i.e. students in the same tutoring group are not independent. By employing the lavaan.survey package, which is designed to deal with non-independently and identically distributed samples [23], we were able to take this clustering (or tutor) effect into account when analyzing the data.

### Ethical approval or consent

No formal ethical approval was sought. Informed consent was given by the participants and participating to the study (i.e. questionnaires) was voluntary. Participating in the PBL-groups was part of the regular curriculum for students. The study had no consequences for grading and did not jeopardize equal opportunities for learning. The research has nothing to do with the student’s assessment and confidentiality was maintained throughout the research. No student was identified at any stage.

## Results

### Response rate and reliability

Data were gathered during the academic years 2011–2012 and 2012–2013, meaning that two cohorts of students are involved. In total, 333 questionnaires of students could be linked to their achievement results: 188 students (out of the 260, i.e. a response rate of 72%) in the first academic year (cohort 1) and 145 students (of the 220, i.e. a response rate of 70%) in the second academic year (cohort 2). Cohort 1 filled in questionnaires about 14 tutors and cohort 2 filled in questionnaires about 12 tutors. Due to this, students of 26 tutorial groups (out of the 32, i.e. response rate of 81%) answered the questionnaire. The data were therefore collected for 16 different tutors. The average number of students completing the instrument per tutorial group was 13 (*SD* = 1.81, range 8–16). A prerequisite for validity is that minimum six students answer the instrument for one tutor [11]. This criterion was met for all tutorial groups.

### Model testing

Because we used an extra item to measure the factor group functioning, Cronbach’s alpha was calculated for this factor. It has a coefficient of 0.896 (*n* = 327, 6 excluded), which means that there is a good internal consistency of the scale. In addition, a confirmatory factor analysis indicated that the present (full theoretical) model was adequate.

**Figure 2:**
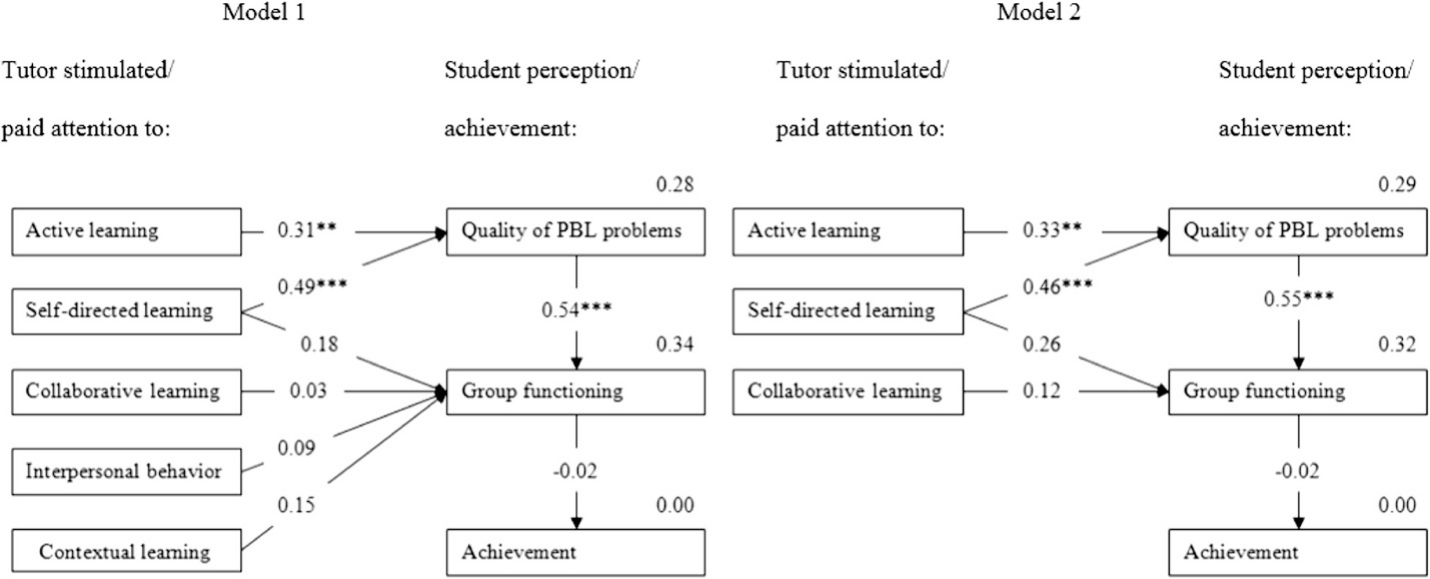
Theoretical model (model 1) and simplified theoretical model (model 2) of correlation between PBL characteristics as perceived by two cohorts of third year medical students in a hybrid curriculum. *Note*. **p ≤ 0.01, ***p ≤ 0.001.

In a first step, the full theoretical model (model 1) and the simplified theoretical model (model 2) of van Berkel and Dolmans were tested [5]. Figure 2 shows the path coefficients or beta weights which symbolize the effects of the independent variables (active, self-directed, collaborative and contextual learning, interpersonal behavior) on the dependent variables (quality of PBL problems, group functioning and achievement) and the squared multiple correlations (R^2^) for the dependent variables. The fit of both models is reported in Table 3.

**Table 3:** **Fit indices of the tested SEM models**

	Value indicating model fit	Model 1	Model 2	Model 3
Number of free parameters		65	44	32
Chi-square		113.793	52.759	33.162
d.f.		87	46	22
*P*-value (Chi-square)	>0.05	0.029	0.229	0.060
CFI	>0.95	0.987	0.995	0.989
TLI	>0.95	0.982	0.992	0.981
RMSEA	<0.05	0.031	0.021	0.039
SRMR	<0.06	0.029	0.028	0.028

**Figure 3:**
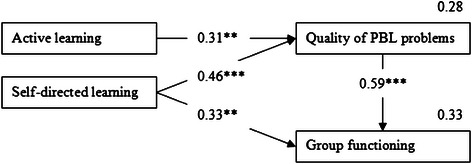
Parsimonious model (model 3) of correlation between PBL characteristics as perceived by two cohorts of third year medical students in a hybrid curriculum*. Note*. *p ≤ 0.05, **p ≤ 0.01, ***p ≤ 0.001.

Based on the R^2^ of achievement and on the beta weights, there seems to be room for improvement of the model. The direct effect of collaborative learning on group functioning is rather small and not significant. In addition, the group functioning is not a significant predictor for achievement. Therefore we looked for a more parsimonious model (model 3, see Figure 3) without the factor “(did the tutor stimulate) collaborative learning” and without the dependent variable “achievement”.

There were only small differences between the coefficients and the squared multiple correlations between model 2 and 3. This implies that the removal of the independent variable “collaborative learning” and the dependent variable “achievement” neither changed the squared multiple correlations nor the beta weights. The effect of self-directed learning on group functioning is now larger and significant. Table 3 shows the comparison between the values of the fit indices resulting from testing the three models. The three models yield good fits of the models to the data.

## Discussion

The aim of this study was to examine which tutor characteristics are the most important in the process of PBL environments in a different setting than the one from which the model of van Berkel and Dolmans was deduced [5]. More specifically, in our study PBL was organized with a larger number of students per tutorial group and within a more hybrid curriculum. We found that stimulating active and self-directed learning are perceived as tutors’ most important tasks with regard to problem quality and group functioning. We expected some differences due to the different nature of the hybrid curriculum. Three main differences are discussed. In our study, (a) stimulating collaborative learning was found not to be as important, (b) stimulating self-directed learning was found to be more important, and (c) there was a larger effect between the perception of problem quality and the perceived group functioning in our study. On the other hand, we found no effect of perceived group functioning on achievement. We now discuss these differences in detail.

The first difference, namely the finding that stimulating collaborative learning was not so important in our study, could be explained by the fact that when the tutor stimulates students less toward collaborative learning, students do not experience this as a shortcoming, as long as the tutorial group does its work [19]. Other authors [25] mention that the extent of learning in PBL does not result from either group collaborations or individual knowledge acquisition in isolation: both activities contribute equally to learning in PBL. This can explain why stimulating collaborative learning was found not to be so important for the perceived functioning of the group.

The second difference is that the impact of self-directed learning on the perceived case quality is much stronger (beta weight = 0.46) than in the previous study of van Berkel and Dolmans (β = 0.25), even when comparing exactly the same two models (cf. model 2 in Figure 2). Combined with the fact that self-directed learning directly impacts on group functioning, and indirectly by the strong effect of the perceived case quality on the group functioning, those results indicate that it is important for the tutor to stimulate self-directed learning in students. It seems obvious that stimulation of self-directed learning by students must be encouraged. In this respect, tutors should be trained to help students to develop critical thinking skills, metacognitive thinking and self-directed learning strategies [26] because students need support and guidance to foster the development of self-directed learning [27]. Tutors can promote self-directed learning directly by teaching learning strategies or indirectly by arranging a learning environment that enables students to practice self-directed learning [28]. However, consistency in interpretation of key concepts like self-directed learning is also an important factor in the success of PBL curricula [29]. Another possible explanation for this finding is the experience of students with PBL (and thus self-directed learning). One of the key characteristics of PBL is the own responsibility of the learners to be self-directed and self-regulated in their learning [4]. In the hybrid curriculum, students have less experience with PBL than in a full PBL curriculum. In this respect, students in the hybrid curriculum may need more support from the tutor to generate clear learning objectives by themselves and to search for various resources by themselves.

The third difference is that we found no effect of group functioning on the achievement score of the students. This may be explained by different insights into learning, group functioning and student performance. First, researchers argue that the scores students give themselves (or in this case to the group), are not always valid [30,31]. Other authors confirmed this issue: they observed that students perceived their groups as “working well as a team”, but observers noted that several aspects of group productivity were not addressed [21]. Students may have judged the group functioning to be adequate given the large groups, but still had too few opportunities to elaborate in detail, which in turn can explain that there was no effect of group functioning on student achievement. A second explanation for the absence of an effect on achievement, could be the period of self-study between the last tutorial session and the exam. Although the exam is taken on the long term and focuses on measuring the application of knowledge, two issues that are in line with PBL as PBL is focusing on applying knowledge and is recommended for enhancing knowledge on the long term, the exam cannot be seen as a direct measure of students’ activities in the tutorial groups. Since there is a period of self-study after the PBL tutorials in which students can prepare for the exams, students can compensate for weak tutorial experiences by studying hard and this can explain why we found no direct effect on the achievement score. A third explanation also emphasizes the individual learners, more specifically in relation to the learning environment. Each learner (with different learner characteristics) interprets the learning environment in a different way, which implies that each learner uses the learning environment in the way that suits their own preferences of learning the best [32-34]. This implies that (a) learners have a different interpretation of good group functioning and (b) some students have more benefits of a well-functioning group than others. Finally, a fourth explanation, is based on the finding that when there is a knowledge conflict, students in tutorial groups lack good argumentation skills and may not be able to engage in collaborative elaboration of conflicting ideas [35]. This may explain the absence of an effect of group functioning on the achievement score.

One of the limitations of this study is that the dataset is relatively small. A second limitation is that a halo effect may have occurred in the responses of the students [21,36,37]. It can be difficult for students to evaluate each aspect of PBL separately from others, which can lead to less variance in the answers of the students. Third, the study used indirect methods such as questionnaires and multiple-choice exams, rather than direct video recording of tutorials. We must note that all variables are perceived variables measured by student questionnaires at the same time (at the end of the academic year). Although this approach is similar to earlier studies and existing theoretical models are used, we need to be cautious when interpreting these results. Future research could focus on data triangulation to complement the self-report measures on the one hand, and gathering multiple measurements (e.g. tutorial observations) throughout the PBL process on the other hand. A fourth limitation is that the tutorial groups exist of about 16 students. The groups are in other words larger than recommended elsewhere [8], but group engagement and participation of students in the discussion is guaranteed (and scored) by the tutor, so the possibility to collaborative learning is assured. Finally, there are still many lectures in the curriculum next to the tutorial sessions, so students may still rely heavily on the lectures.

In future research, tutor characteristics should be examined further and investigation must show how important tutor tasks can be trained. For example, it can be useful to investigate how active and self-directed learning can be promoted by tutors. Therefore, future studies should focus on what actually happens in a PBL session that constitutes the stimulation of active learning and self-directed learning, by e.g. direct observation of the learning environment. In addition, other instruments aiming at directly measuring group functioning may be applied to complement the self-report measures.

## Conclusions

The added value of this research is that this study is conducted in a hybrid PBL curriculum instead of a full PBL curriculum. This study is also important with respect to its specific methodology, in which the data were pooled for the tutoring group when analyzing the model through structural equation modeling.

In our study, a large effect between the perception of problem quality and the perceived group functioning was found. On the other hand, we found no effect of perceived group functioning on achievement, and the stimulation of collaborative learning was found to be less important. The most important findings were that stimulating active and self-directed learning are perceived as tutors’ most important tasks with regard to problem quality and group functioning (in a hybrid curriculum). As a practical implication, tutors should be trained to promote these skills in students.

## Abbreviations

PBL: Problem-based learning
SEM: Structural equation modeling
